# Greenhouse gas observations from the Northeast Corridor tower network

**Published:** 2020

**Authors:** Anna Karion, William Callahan, Michael Stock, Steve Prinzivalli, Kristal R. Verhulst, Jooil Kim, Peter K. Salameh, Israel Lopez-Coto, James Whetstone

**Affiliations:** 1 Special Programs Office, National Institute of Standards and Technology, Gaithersburg, MD, USA; 2 Earth Networks, Inc., Germantown, MD, USA; 3 NASA Jet Propulsion Laboratory, California Institute of Technology, Pasadena, CA, USA; 4 Scripps Institution of Oceanography, University of California San Diego, La Jolla, CA, USA; 5 Engineering Laboratory, National Institute of Standards and Technology, Gaithersburg, MD, USA

## Abstract

We present the organization, structure, instrumentation, and measurements of the Northeast Corridor greenhouse gas observation network. This network of tower-based in situ carbon dioxide and methane observation stations was established in 2015 with the goal of quantifying emissions of these gases in urban areas in the northeastern United States. A specific focus of the network is the cities of Baltimore, MD, and Washington, DC, USA, with a high density of observation stations in these two urban areas. Additional observation stations are scattered throughout the northeastern US, established to complement other existing urban and regional networks and to investigate emissions throughout this complex region with a high population density and multiple metropolitan areas. Data described in this paper are archived at the National Institute of Standards and Technology and can be found at https://doi.org/10.18434/M32126 (Karion et al., 2019).

## 1 Introduction

As the population of cities grows globally due to trends toward urbanization, so does their relative contribution to global anthropogenic greenhouse gas (GHG) budgets (Edenhofer et al., 2014; O’Neill et al., 2010). City governments are making commitments to reduce their emissions of GHGs through various sustainability and efficiency measures and coordination with organizations like the C40 Climate Leadership Group (http://www.c40.org, last access: 23 March 2020), the Global Covenant of Mayors for Climate and Energy (https://www.globalcovenantofmayors.org, last access: 23 March 2020), and others. These organizations require individual cities to conform to certain standardized mechanisms and practices for reporting their carbon emissions. City governments rely on inventories compiled using data on fuel use, energy usage, etc., to estimate their total emissions and changes over time and to determine the efficacy of various emissions mitigation policies. Analysis of atmospheric measurements provides additional useful information to such efforts, by confirming inventory estimates (Sargent et al., 2018; Lauvaux et al., 2016), detecting trends (Mitchell et al., 2018), or estimating emissions that are not well quantified using inventory methods, such as methane emissions (McKain et al., 2015; Ren et al., 2018; Lamb et al., 2016; Yadav et al., 2019). Several urban top-down measurement efforts are underway in various cities that include networks of observations, often in situ CO_2_ and CH_4_ measurements from rooftops or towers (Verhulst et al., 2017; Xueref-Remy et al., 2018; Bares et al., 2019) or using other long-path and remote sensing methods (Waxman et al., 2019; Hedelius et al., 2018; Wong et al., 2016; Pillai et al., 2016).

The National Institute of Standards and Technology (NIST) has partnered with other federal, private, and academic institutions to establish three urban test beds in the United States: the Indianapolis Flux Experiment (INFLUX, http://influx.psu.edu, last access: 23 March 2020), the Los Angeles Megacities Carbon Project (http://megacities.jpl.nasa.gov, last access: 23 March 2020), and the Northeast Corridor (NEC, http://www.nist.gov/topics/northeast-corridor-urban-test-bed, last access: 23 March 2020). The goals of the urban test beds are to develop and refine techniques for estimating greenhouse gas emissions from cities and to understand the uncertainty of emissions estimates at various spatial and temporal scales (e.g., whole city annual emissions vs. 1km weekly emissions). Recent results from the longest-running test bed, INFLUX, show that whole city emissions can be estimated using three different methods to within 7% (Turnbull et al., 2019).

The Northeast Corridor (NEC) was established in 2015 as the third NIST urban test bed. The goals for this project are to demonstrate that top-down atmospheric emissions estimation methods can be used in a domain that is complicated by many upwind and nearby emissions sources in the form of surrounding urban areas. The objective is to isolate the anthropogenic GHG emissions from urban areas along the US East Coast from many confounding sources upwind (cities, oil and gas development, coal mines, and power plants) and from the large biological CO_2_ signal from the highly productive forests nearby and within the cities. The presence of highly vegetated areas such as urban parks, local agriculture, and managed lawns is expected to dominate the CO_2_ signal in summertime, as has been found in Boston, MA (Sargent et al., 2018). The NEC project has a current focus on the urban areas of Washington, DC, and Baltimore, MD, USA, with existing plans to expand northward to cover the entire urbanized corridor of the northeastern US, including the cities of Philadelphia and New York City, and eventually linking up with existing measurement stations in Boston, MA (McKain et al., 2015; Sargent et al., 2018).

The NEC project includes multiple measurement and analysis components. The backbone of the NEC project is a network of in situ CO_2_ and CH_4_ observation stations with continuous high-accuracy measurements of these two greenhouse gases. In addition, periodic flight campaigns of multiple weeks each year are conducted by the University of Maryland (FLAGG-MD, http://www.atmos.umd.edu/~flaggmd, last access: 23 March 2020) and Purdue University (https://www.science.purdue.edu/shepson/research/ALARGreenhouseGas/, last access: 23 March 2020), focusing on wintertime observations of CO_2_, CH_4_, CO, O_3_, SO_2_, and NO_2_ from instrumented aircraft (Ren et al., 2018; Salmon et al., 2018; Lopez-Coto et al., 2020a). The use of low-cost CO_2_ sensors is also being investigated in Washington, DC, with work focusing on calibration and determination of long-term stability of inexpensive nondispersive infrared (NDIR) sensors with potential for use in CO_2_ data assimilation techniques (Martin et al., 2017). The NEC project also includes an extensive modeling component. First, high-resolution meteorological modeling (using the Weather Research and Forecast (WRF) model) is being conducted (Lopez-Coto et al., 2020b), with output coupled to Lagrangian dispersion models such as STILT (Lin et al., 2003; Nehrkorn et al., 2010) and HYSPLIT (Stein et al., 2015). These transport and dispersion models are used to interpret observations from both aircraft and tower stations and in atmospheric inverse analyses to estimate fluxes of CO_2_ and CH_4_ from the cities of Washington, DC, and Baltimore, MD (Lopez-Coto et al., 2020a; Huang et al., 2019). A high-resolution fossil fuel CO_2_ inventory, Hestia, is also being developed for this project (Gurney et al., 2012, 2019).

Here we focus on the high-accuracy tower observation network and associated data collection and processing methods. Section 2 describes the tower network design and characterizes the different site locations; Sect. 3 describes the measurement methods, instrumentation, and calibration; Sect. 4 presents the uncertainty derivation for the measurements; and, finally, Sect. 5 presents some of the observations from the current record.

## 2 Network design and site characterization

**Figure 1.**
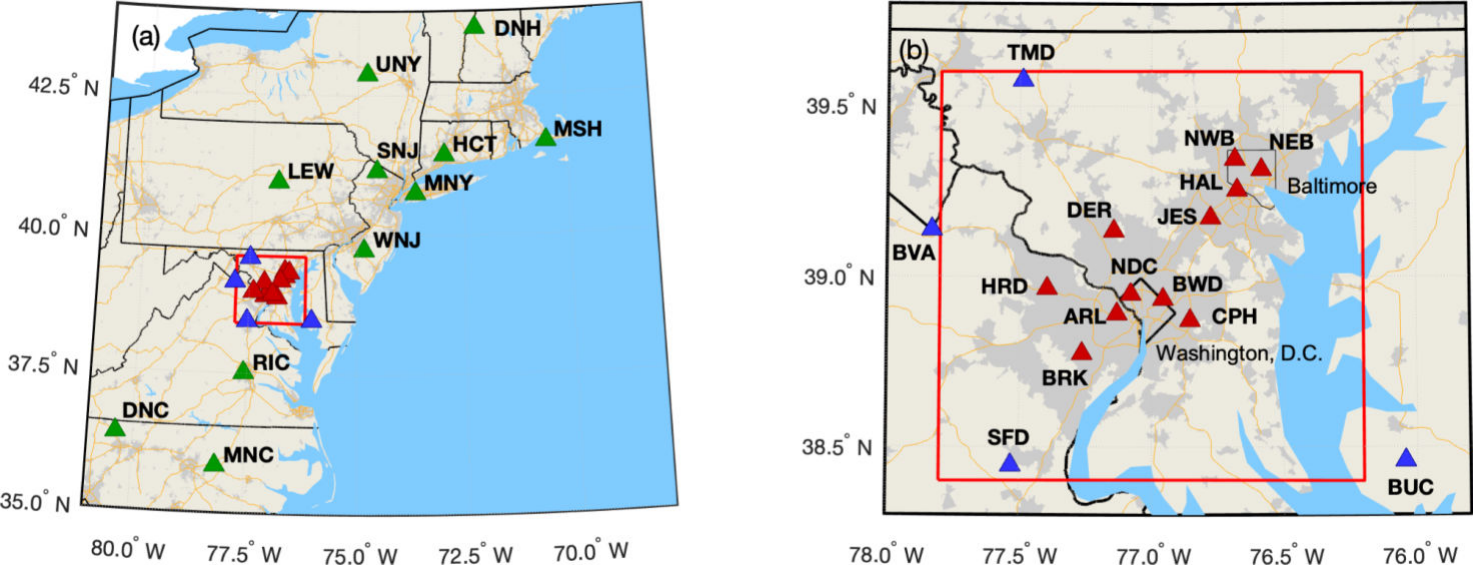
Locations of Northeast Corridor (NEC) established tower-based observations, corresponding to Table 1. The red rectangle indicates the modeling analysis domain. Light grey shading indicates census-designated urban areas, yellow lines are interstate highways, and black boundaries are state lines, with a thinner black line showing the City of Baltimore. Green triangles indicate regional sites, red triangles indicate urban sites, and blue triangles are more rural or background sites surrounding the Washington–Baltimore domain. (**a**) Regional map. (**b**) Inset focusing on Washington, DC, and Baltimore, MD. All map data layers were obtained from either Natural Earth (http://naturalearthdata.com, last access: 23 March 2020) or U.S. Government sources (http://www.census.gov, last access: 23 March 2020) and are in the public domain.

The NEC project includes 29 observation stations, all managed and operated by Earth Networks, Inc.^1^ (http://www.earthnetworks.com/why-us/networks/greenhouse-gas, last access: 23 March 2020). A total of 10 stations were existing Earth Networks (EN) measurement sites in the northeastern US that became part of the NEC project in 2015. A total of 19 stations were established (or will be established) specifically for the NEC project, with site locations identified by NIST. A total of 16 of these station locations were chosen to be used for emissions estimation in a domain around Baltimore and Washington, DC (red boundary, Fig. 1), using inverse modeling techniques (Lopez-Coto et al., 2017; Mueller et al., 2018). Three others are in Mashpee, MA, Philadelphia, PA, and Waterford Works, NJ. As of publication, 14 of these 19 have been established, with delays occurring due to difficulty finding suitable tower locations to agree to house the systems. The hardware and software operating at all the sites is identical with few exceptions as noted in the text.

The initial design of the core urban Baltimore–Washington network was focused on optimizing tower site locations with the goal of reducing uncertainty in estimating anthropogenic CO_2_ emissions from Washington, DC, and Baltimore using an atmospheric inversion model (Lopez-Coto et al., 2017). A total of 12 communications towers were identified as part of that study as ideal locations for measurements. Actual measurement sites were sometimes established at locations near the ideal study location, usually due to logistical difficulties obtaining leases at the ideal tower sites. A second design study determined ideal locations for background stations, i.e., observation station locations that would aid in the determination of background CO_2_ entering the analysis domain (Mueller et al., 2018). Four stations were identified as part of that study; an existing EN site in Bucktown, MD, serves as a fifth background station southeast of the analysis domain (Fig. 1). Although inlet heights were desired to be 100m above ground level (a.g.l.), often shorter towers were used due to the lack of availability of tall towers in ideal locations; the shortest tower in this network has the uppermost inlet at 38ma.g.l. (HRD). Table 1 indicates details and locations of each site.

**Figure 2.**
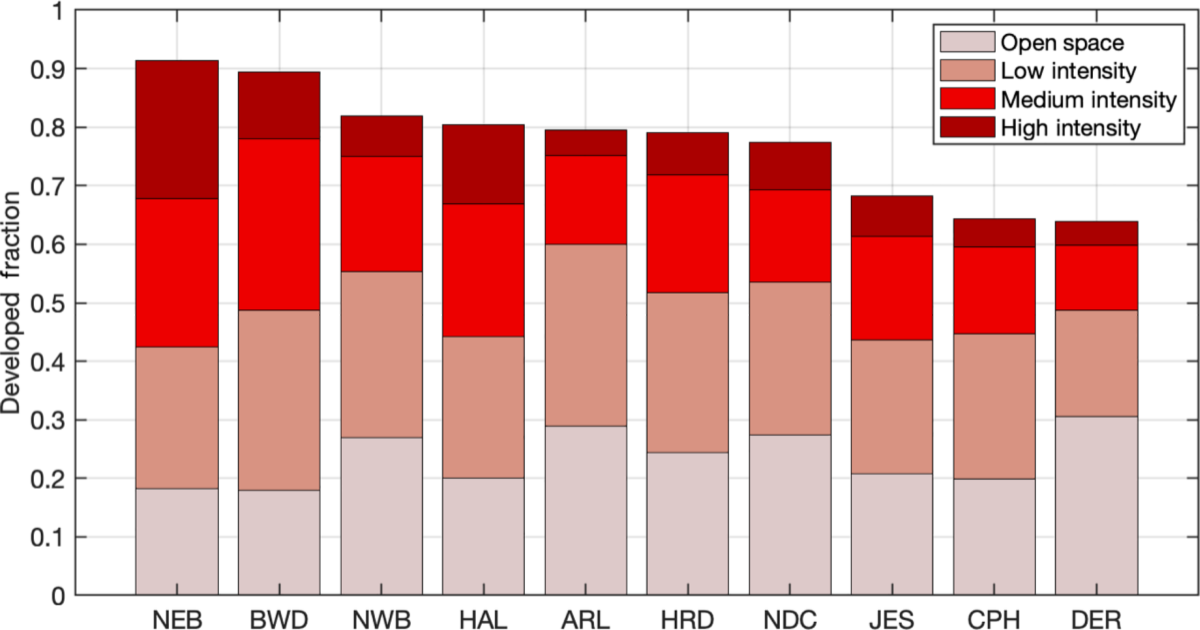
Fraction of developed land cover (from the 2016 National Land Cover Database (NLCD); Yang et al., 2018) within 5 km of each observation station in the urban regions of Washington, DC, and Baltimore, MD.

The stations in Table 1 are all situated in areas with different land use. Even among the Washington, DC, and Baltimore area urban stations, the degree of urban intensity varies, from densely urbanized areas (such as northeastern Baltimore, NEB) to dense and moderately developed suburbs (such as Capitol Heights, CPH, and Derwood, DER, both suburbs of Washington, DC, located in Maryland). Figure 2 indicates the intensity of development from the US Geological Survey (USGS) 2016 National Land Cover Database (Yang et al., 2018) surrounding each urban station in the Washington, DC, and Baltimore network.

**Figure 3.**
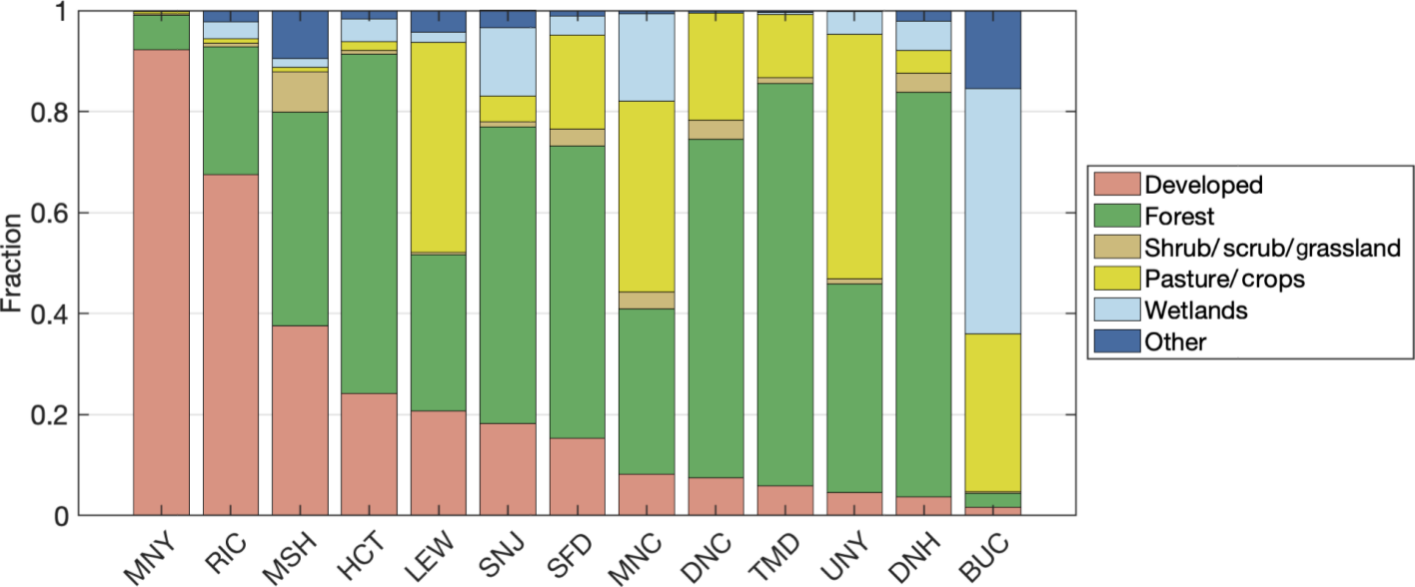
Average fraction of land cover type within 5 km of regional tower sites in the Northeast Corridor network, in order of decreasingly developed land. Several NLCD classifications have been grouped for clarity (e.g., “developed” includes open spaces and low-, medium-, and high-intensity developed land). SFD, TMD, and BUC are sites established to help characterize background conditions for the Washington, DC, and Baltimore urban network.

Similar variability in land cover for the regional stations exists, as indicated in Fig. 3. The sites established to characterize background conditions for the urban network in Washington, DC, and Baltimore (SFD, TMD, BUC) are in areas with little development: SFD and TMD are both in forested regions, while BUC is near the Chesapeake Bay and large wetland areas. The other regional sites span a range of land cover types from urban (MNY in New York City and RIC in Richmond, VA), to mostly rural and forested (DNH in Durham, NH).

## 3 Carbon dioxide, methane, and carbon monoxide measurements, instrumentation, and calibration

### 3.1 Instrumentation

**Figure 4.**
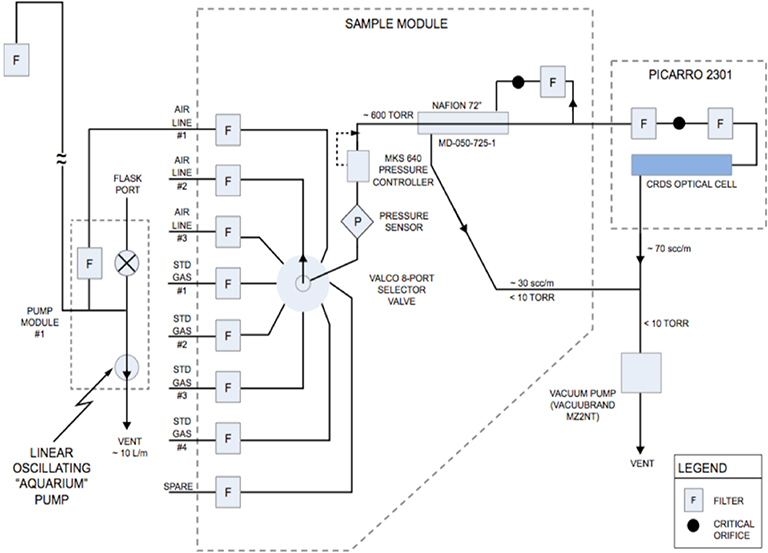
Plumbing diagram for the Earth Networks sampling system implemented at the NEC tower stations. Figure replicated from Fig. S1 in Verhulst et al. (2017), adapted from Welp et al. (2013).

The instrumentation contained in the Earth Networks (EN) system module has been described elsewhere (Welp et al., 2013; Verhulst et al., 2017); we will summarize the system here but refer the reader to those publications for further details, including additional equipment and part numbers. Figure 4 indicates the plumbing diagram of the typical tower setup. Three inlet lines reach from the sampling location on the tower into the equipment housed in a full-size rack inside a shed at the base of the tower. Typically, two inlet lines sample from the topmost level and one line samples from a lower level on the tower. Stafford, VA (SFD), is one exception with inlets at three different levels (50, 100, and 152m), and a planned tower in New Jersey (Waterford Works) will have five inlet height levels, as indicated in Table 1. At some sites there was no space to house the equipment in existing structures, thus small single- or double-rack sized enclosures were purchased and installed. Air is pulled through a filter into the inlet lines (0.953cm, i.e., 0.375in., OD Synflex 1300) that are continuously flushed at ~ 10 L min^−1^ by aquarium pumps (Alita AL-6SA). The three air lines are connected to a rotary multi-port valve (MPV; eight-port, VICI, Valco Instruments Co. Inc.) housed within a sample control box (calibration box). Two or three calibration standards are also connected to the MPV with 0.156 cm (0.0625 in.) OD stainless steel tubing. The control system for the MPV directs the air stream to the analyzer cycling every 20 min through each of the three inlet lines so that each inlet is sampled at least once an hour and every 22h through each standard (Sect. 3.2). The common port of the MPV is connected to a pressure controller that reduces the pressure to 80 kPa (800 mb), after which the sample (either ambient air or air from a standard gas cylinder) enters a 183cm long Nafion dryer (Permapure, Inc., model MD-050–72S-1), where it is dried to a water vapor mole fraction of ~ 0.1% prior to flowing through the cavity ring-down spectroscopic (CRDS) analyzer (Picarro, Inc., Model 2301). The lower-than-ambient inlet pressure of 80 kPa is prescribed in order to lower the flow rate of the analyzer to ~ 70standard cm^3^ min^−1^. At Mashpee, MA (MSH), a CRDS Picarro Model 2401 analyzer is operational, and this is the only site currently also measuring carbon monoxide (CO) in addition to CO_2_ and CH_4_. The CRDS analyzers report measurements of dry air mole fraction of each gas in air, also known as the mole fraction, i.e., moles of the trace gas per mole of dry air. Throughout, we refer to these measurements in units of μmol mol^−1^ for CO_2_ and nmol mol^−1^ for CH_4_ and CO, following the SI recommendations (Bureau International des Poids et Mesures, 2019). Software (GCWerks, Inc.) installed on a separate mini-PC at each site controls the run cycle and the MPV selection valve. The data are collected on this computer and sent to the central EN data server, also running GCWerks. All data are processed on the central EN data server but additional post-processing and uncertainty assignment to hourly observations is performed at NIST. As recommended by the World Meteorological Organization (WMO), the software has the capability of reprocessing all the data from the original raw files and thus can accommodate any changes to the assigned values of the standards (due to a reference-scale update, for example) at any time (WMO, 2018).

### 3.2 Calibration cylinders

When the Earth Networks GHG monitoring system was established in 2011, each site hosted two calibration cylinders (standards) with ambient level dry air mole fractions as part of the original system design. This continues to be the case at most NEC sites. At the NEC sites, these standards have values close to 400 μmol mol^−1^ dry air CO_2_, 1890 nmol mol^−1^ dry air CH_4_, and 115 nmol mol^−1^ dry air CO (at MSH only) and are sampled by the analyzer periodically, in a sequence identical to that described for the Los Angeles Megacity network by Verhulst et al. (2017). The standards are purchased from the WMO Central Calibration Laboratory (CCL), the National Oceanographic and Atmospheric Administration’s Earth System Research Laboratory (NOAA/ESRL) Global Monitoring Division in Boulder, CO, USA, where they have been calibrated on the WMO scales (X2007 for CO_2_, X2004A for CH_4_, and X2014A for CO, Zhao et al., 1997; Dlugokencky et al., 2005; Novelli et al., 2003). One of these two cylinders serves as a standard for calibration and drift-correction, while the second serves as a target tank or check standard. The target tank is used for data quality checks and uncertainty calculations (Sect. 4). The residual of the target tank (the rms difference between its value assignment when treated as an unknown and its reference value from NOAA) is a critical indicator of data quality and is monitored in order to alert the operators of any general problems in the system such as leaks, mistakes in the assignment of MPV ports, or drift in calibration tank value. In the field, all gas standards are sampled for 20 min every 22h. In data processing, the first 10 min of any tank run are filtered out to allow for the system equilibration, including flushing of the regulator and tubing. In some cases, when the standard runs were found not to equilibrate as quickly as desired, 15 min of data were filtered until the problem could be fixed (typically either contamination or inadequate regulator flushing). The first 10 min of the ambient air sample following a standard run are also filtered for equilibration, and the first 1 min of each 20 min ambient air run is filtered if it follows another ambient air run (i.e., an inlet switch). The longer flush time is desired for the standard runs because of the need to flush stagnant air remaining in the regulators and tubing when sampling from the cylinder, while the ambient air lines are continuously flushed.

At a few NEC sites (currently BWD and MSH, with more planned), a third gas cylinder is installed at the site to serve as a permanent high-concentration standard (referred to as the high standard), to improve calibration and reduce uncertainties. This standard typically contains air with a mole fraction of CO_2_ close to 500 μmol mol^−1^, CH_4_ at approximately 2300 to 2500 nmol mol^−1^ and at MSH, CO, near 320 nmol mol^−1^. At MSH, this cylinder has been provided directly by NOAA/ESRL, while at BWD this cylinder was purchased as natural whole air from Scott-Marrin, Inc. (now Praxair). The Scott-Marrin air is stripped of its original trace gases (CO_2_, CH_4_, CO, hydrocarbons, etc.) with CO_2_, CH_4_, and CO added back in to prescribed values. Several such standards have been purchased with the intent of placing them at urban stations to serve as high standards after calibrating them onto the WMO scales. We note that because they are being used together with NOAA/ESRL standards in the field, it is essential that these standards also be assigned values on the same scales. This calibration is transferred in the NIST laboratory using five standards calibrated and purchased from NOAA/ESRL. The CO_2_ in the Scott-Marrin cylinders is isotopically different (in terms of the ^12^C*/*^13^C ratio in CO_2_) from the ambient air tanks that are filled by NOAA/ESRL at Niwot Ridge, CO. However, the calibration is transferred from the NOAA standards to the Scott-Marrin gases using the same model (Picarro 2301) analyzer used in the field (i.e., measuring only ^12^CO_2_) in the NIST laboratory, effectively canceling out the error that would be caused by this isotopic mismatch (Chen et al., 2010; Santoni et al., 2014). Thus, the CO_2_ values assigned by NIST to these standards are effectively the total dry air mole fraction of CO_2_ the cylinders would contain if they were isotopically similar to the NOAA cylinders.

Additional sites in the network also benefit from the improved two-point calibration method in cases where measurements of a high standard were performed prior to analyzer deployment (NWB, NEB, JES, TMD, CPH, and HRD). Prior to system installation at these sites, tests were conducted at the EN laboratory in which the designated analyzer was set up measuring the calibration standard, target standard, and a high-value standard at ~ 490 μmol mol^−1^ CO_2_ and ~ 2560 nmol mol^−1^ CH_4_ daily for several days (enough for 3–5 measurements of 20 min each). This single high-standard cylinder was also calibrated by and purchased from NOAA/ESRL, with assigned values on the WMO scales. These laboratory tests allow the determination of the secondary correction to the instrument response or sensitivity, as described in Sect. 3.4.

The high-standard gas measurements are used to perform a secondary correction (referred to as a two-point calibration) (Sect. 3.4) to the original one-point calibration described by Verhulst et al. (2017) and in Sect. 3.3., reducing the uncertainty of the measurements. We note that while, in principle, a secondary correction is desirable, and the uncertainty is indeed reduced by its implementation (see Sect. 4.2), it remains quite small relative to the signals of interest in an urban network. Deployment of high standards at all sites has not yet occurred due to both costs and logistical and operational constraints; for example, at many sites the space available for the equipment is limited and prohibits the installation of a permanent third tank. Thus, we plan to implement a round-robin procedure circulating additional standards at various values through the network to evaluate the calibrations and implement the secondary correction throughout the network. Although the current state of having two different calibration methods coexisting in the network is not ideal, we aim to implement the secondary correction throughout the network as soon as possible.

### 3.3 Drift correction and single-point calibration

Here we describe the calibration and drift correction applied to all the mole fraction data. This single-point calibration uses only a single reference value, that of the calibration standard, to correct the raw mole fractions for each gas. The equations are identical (with a few nomenclature differences) to those found in Verhulst et al. (2017). In the following analysis, *X*′ denotes a raw dry mole fraction measurement (i.e., a reported value from the CRDS analyzer after internal water vapor correction), while *X* denotes a mole fraction after some correction has been applied (drift and/or calibration, as described in the equations below). A subscript cal indicates the main calibration standard (usually a single ambient level standard tank calibrated by NOAA/ESRL), subscript std indicates any other standard tank, tgt indicates a standard tank that is being used as a target, and the subscript air indicates the sample measurement. Note that within the GCWerks software, the meanings of the abbreviations cal and std are reversed from what is defined here; we choose to use the nomenclature by Verhulst et al. (2017) here for consistency with the literature. We note that we have changed some nomenclature slightly from Verhulst et al. (2017) for additional clarity and conciseness. We refer to the drift-corrected mole fraction as *X*_DC_, which is noted as *X*_corr_ by Verhulst et al. (2017); we refer to the mole fraction after a secondary correction is applied as *X*_SC_. We also refer to the assigned mole fraction of a standard by the calibration laboratory as *C* rather than *X*_assign_. We define the sensitivity *S* to be the response of the analyzer or the ratio of the measured to the true value. In the case of the calibration tank, this is the ratio of the raw measured value, $X_{\text{cal}}^{\prime }$, to the assigned value of the standard by the calibration laboratory on the WMO scale for the given species, *C*_cal_:

(1) $$S=\frac{X_{\text{cal}}^{\prime }}{C_{\text{cal}}}.$$

When only a single calibration standard is present (which is the case at most sites in the NEC network), this sensitivity is assumed to be constant across mole fractions but varying in time. The sensitivity for the calibration tank is thus interpolated in time and applied as a correction for the dry air mole fractions of CO_2_ and CH_4_ reported by the CRDS analyzer $\left(X_{\text{air}}^{\prime }\right)$:

(2) $$X_{\text{DC},\text{air}}=\frac{X_{\text{air}}^{\prime }}{S},$$

where *X*_DC,air_ is the drift-corrected air data. An alternative drift-correction is to use an additive offset, which is also interpolated in time, rather than a sensitivity for drift correction:

(3) $$X_{\text{DC},\text{air}}=X_{\text{air}}^{\prime }+\left(C_{\text{cal}}-X_{\text{cal}}^{\prime }\right).$$

**Figure 5.**
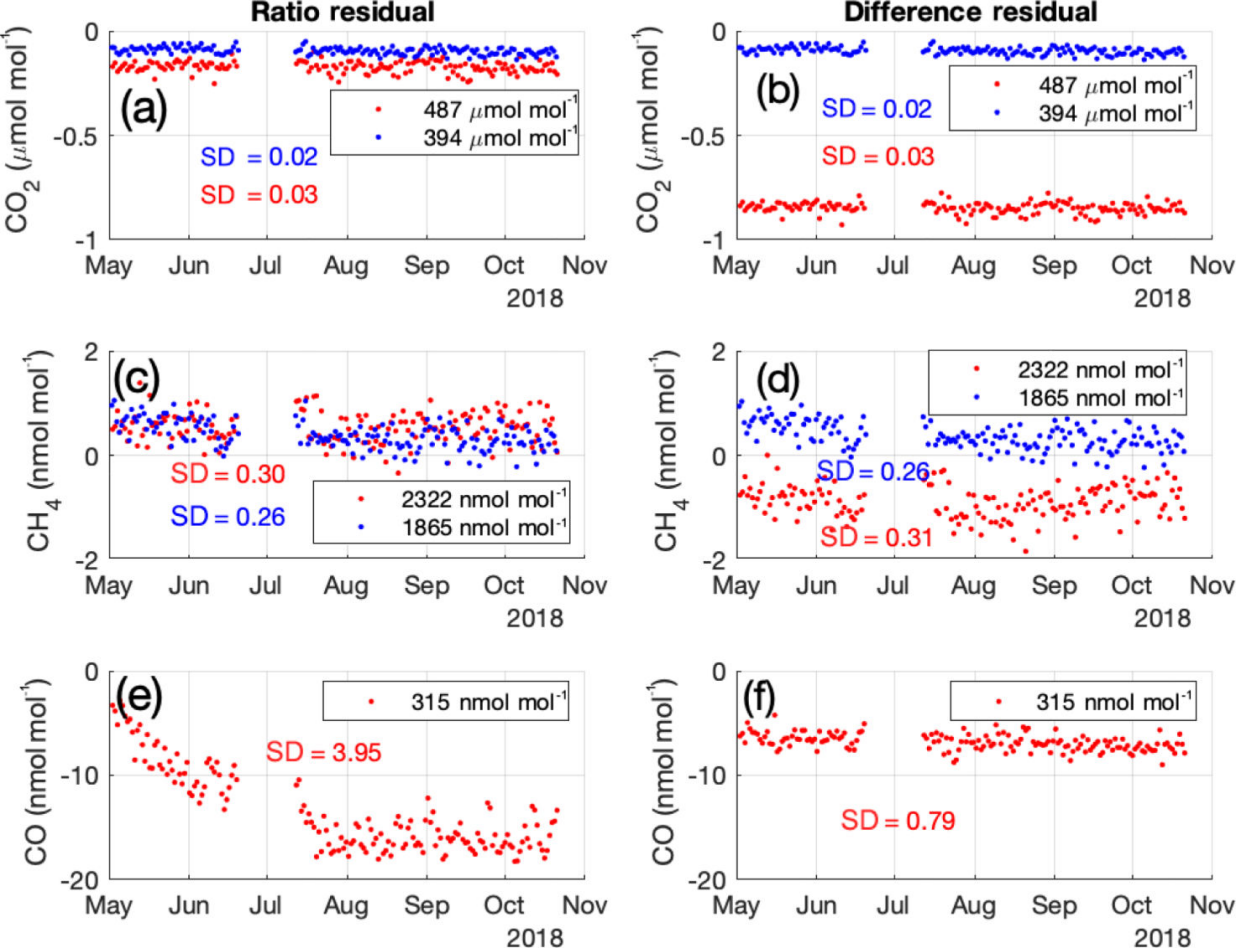
Time series of standard tank run residuals (i.e., *X*_DC_−*C*) for CO_2_ (**a, b**), CH_4_ (**c, d**), and CO (**e, f**). *X*_DC_ is calculated using a single calibration tank (not shown) and the ratio method (Eq. 2) on the left (a, c, e) and the difference method (Eq. 3) on the right (**b, d, f**). Assigned tank values are shown in the legend; one tank was not calibrated for CO so only the residuals of the high-concentration tank at 315 nmol mol^−1^ are shown. The residual magnitude is smaller for CO_2_ and CH_4_ using the ratio method, but the standard deviations (variability) are similar using both methods. For CO, both the magnitude of the residual and the standard deviation are smaller using the difference equation; the ratio equation does not properly account for the drift in the analyzer at the start of the time series (May–June). Data shown are from MSH; a measurement gap exists in July.

Measurements from MSH that include a high-value cylinder suggest that the single-tank drift correction performs (very slightly) better using the ratio correction (Eq. 2) than the difference method (Eq. 3) for CO_2_ and CH_4_, while the opposite is true for CO (Fig. 5), thus the difference method is used only for CO in our network.

The calibration standard mole fractions are interpolated in time between subsequent runs in order to apply the above corrections to the air data, thus removing drift in the instrument’s response. This drift-corrected fraction is reported in the hourly data files for sites and time periods where no range of concentrations is available in the standard tanks.

### 3.4 Multiple-point calibration

At some sites and for some time periods, a higher-mole-fraction standard is available, and a second-order correction can be made to the instrument sensitivity, accounting for the sensitivity being a function of mole fraction. Usually in the field, this correction employs only one additional standard, the higher-mole-fraction standard so that it is a two-point calibration; here we describe the general procedure for applying a correction using multiple standards at a range of concentrations. This is applied as a second-order correction to the drift-corrected air data. In general, if a range of standard concentrations is available, the correction in GCWerks is applied as described below. First, a drift-corrected sensitivity (*S*_DC_) is calculated for each standard when it is measured, which is the ratio of the drift-corrected mole fraction of that standard (*X*_DC,std_, based on Eq. 2 for CO_2_ and CH_4_ or Eq. 3 for CO) to its assigned value:

(4) $$S_{\text{DC},\text{std}}=\frac{X_{\text{DC},\text{std}}}{C_{\text{std}}}.$$

For the calibration standard, this value is necessarily equal to 1, but measurements of standard tanks with different assigned values indicate that the instrument sensitivity is dependent on the composition of the sample gas (in this case, the mole fraction of the standard tank). In laboratory calibrations, we find that the drift-corrected sensitivity defined in Eq. (4) is a linear function of the mole fraction ratio to the calibration gas ($X^{\prime }/X_{\text{cal}}^{\prime }$), thus we use a linear fit to the range of standards to determine the slope *m* and intercept *b*:

(5) $$S_{\text{DC}}=m\left(\frac{X^{\prime }}{X_{\text{cal}}^{\prime }}\right)+b.$$

In this fit, we force *m*+*b* = 1 by fitting a slope *m* and then setting *b* = 1−*m* in order to maintain the proper relationship for the calibration tank itself, when *S*_DC,cal_ = 1. Applying this fit to the air data, the final air mole fraction *X*_SC,air_ is determined from

(6) $$X_{\text{SC},\text{air}}=\left(\frac{X_{\text{DC},\text{air}}}{S_{\text{DC}}}\right).$$

In the NEC tower network, there are no sites with multiple standard tanks at various concentrations. At several sites, there are measurements of a single high-concentration standard (hstd) in addition to the calibration and target standards. The high-standard measurements are either performed in the laboratory before the instrument is deployed to the field, or in the field if the third standard is permanently installed (Sect. 3.2). The above secondary correction is applied using only two tanks to perform the fit and obtain the drift-corrected sensitivity. In this special case, the fit has zero degrees of freedom with no residuals. The correction parameters (slope and intercept) are determined based on measurements over time or single measurements in the laboratory prior to a specific analyzer deployment. The correction is applied to the data from the site for a time period that is specified, i.e., it is not automatically applied based on daily measurements of the high standard. It is determined by the science team and applied for the time period that is appropriate. This is necessary to avoid applying the wrong correction if an analyzer is replaced or if there are changes made to the analyzer that might affect its calibration response. At eight sites where a high standard has been measured at any point (MSH, BWD, NWB, NEB, JES, TMD, CPH, and HRD), slopes and intercepts have been determined and the correction has been applied to the data. At stations with no high-standard measurements, we rely on the single-tank drift-correction described in Sect. 3.3.

**Figure 6.**
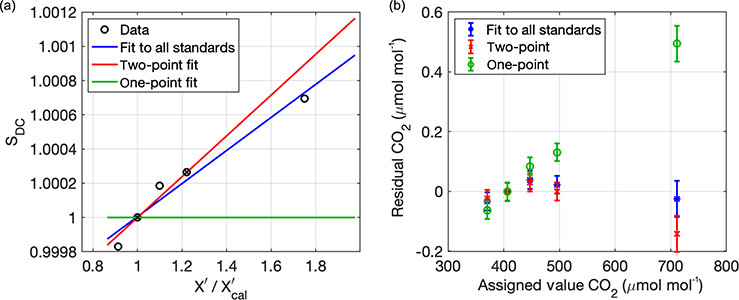
Example of a laboratory calibration of a CRDS analyzer with five standards of different assigned CO_2_ mole fractions. (**a**) Secondary correction of drift-corrected sensitivity using either two (red) or all five (blue) standards. Green line at 1 indicates the assumed sensitivity when only a single standard is used. (**b**) Residual of each type of fit; error bars represent 1*σ* reproducibility stated by NOAA/ESRL. The simple single-tank drift correction results in the green circles as residuals; these residuals were used in the Verhulst et al. (2017) analysis to estimate the extrapolation uncertainty of the single-point correction. Red x symbols are the residuals of a fit to two standards, and blue asterisks are the residuals of the fit to all five standards.

Laboratory tests with multiple standards with the same model instrument used in the network (Picarro 2301) were performed to assess the relative improvement of a fit to two standards over a fit to a single standard. Figure 6a illustrates the fit of the drift-corrected sensitivity (*S*_DC_) to two standards (red line) vs. all five standards (blue line) for CO_2_, along with corresponding residuals in Fig. 6b. As was shown by Verhulst et al. (2017) for multiple analyzers, the fit to a single standard has a linearly varying residual that is typically 0.1 to 0.2 at 100 μmol mol^−1^ above the calibration standard value (green circles, Fig. 6b). The average slope of the one-point residual from multiple tests is used by Verhulst et al. (2017) to estimate the uncertainty of the single-point calibrations (called the extrapolation uncertainty, *U*_extrap_), described in Sect. 4.1. Performing the additional correction using a high standard shows improvement in the residuals of the fit (Fig. 6b), while using all five standards only improves the residuals incrementally. The two-point correction (red) in this figure was applied using the 406 μmol mol^−1^ tank as the calibration and the 496 μmol mol^−1^ tank as the high standard; thus, the measurement at ~ 711 μmol mol^−1^ is an extrapolation of the two-point fit. The residuals at values between the calibration and high standard are very small, equal to or below the uncertainty (reproducibility) of the scale reported by NOAA; this was confirmed for other analyzers and other species.

The improvement in calibration from the secondary correction is quite small compared to the signals and gradients of interest in our network. For example, when considering the enhancement between the rural site TMD and a polluted urban site, HRD, the calibration method makes a median difference of 0.4% for CO_2_ and 0.3% for CH_4_ (over all hours over 1 calendar year). We intend to implement this calibration throughout the network through deployment of additional standards and periodic traveling calibrations when permanent installation is not practical for logistical reasons.

### 3.5 Data quality and processing

Automated data filtering is performed within the GCWerks software with parameters set identically to those extensively described by Verhulst et al. (2017) for the Los Angeles Megacities network. For example, individual measurements that are outside limits for cavity temperature, cavity pressure, and during transitions between sample streams are filtered. The data are automatically downloaded from each site’s Linux PC to the central EN Linux server, where they are processed automatically every hour. We note that all mole fraction assignments can be recalculated by the GCWerks software from the archived raw files if required due to a change in filtering or flagging, or in assignment of a standard tank, for example, in the case of a scale change by the CCL. The data files exported from GCWerks contain 1, 5, and 20 min averaged air data, as well as separate files with 1, 5, and 20 min averages of all standard runs. Individual or groups of 1 min data points are flagged manually by EN or NIST researchers in the GCWerks if there is cause (e.g., a site visit that disrupted the sample stream or a leak in the line). Some additional quality checking is performed at this stage, specifically checking for systematic differences between measurements from two different inlets at the same height and checking for inconsistencies in the difference between measurements at different heights. For example, if the lower inlet is systematically reading lower CO_2_ than the upper inlet, especially at night, it would indicate that the inlet lines may be switched (mislabeled) or there is a leak occurring. These indications would be then verified by a field technician, and the data are either reprocessed or flagged accordingly. Filtered and flagged points are excluded from the subsequent averaging exported by GCWerks. The 1 min air data files and 20 min standard data files are post-processed at NIST to calculate hourly averages from each air inlet level and to assign uncertainties to each hourly average (Sect. 4). Data from the two top-level inlets, when they are at the same height, are combined for inclusion into the hourly average. Thus, because of the 20 min cycling through the three inlets (Sect. 3.1), hourly averages at the upper inlet include approximately 40 min of measurements, and for the lower inlet only 20 min (fewer if a calibration occurs). Publicly released hourly data from this second-level processing are contained in separate files for each species and each level for each site. The files contain the hourly average mole fraction (i.e., mole fraction) along with its uncertainty, standard deviation, and number of 1 min air measurements included in that particular hourly average. These last two quantities are provided so users can determine the standard error of the hourly means in terms of the observed atmospheric variability within the hour. Observations at higher frequency and standard tank data are available by request.

### 3.6 Comparison with measurements of NOAA whole air samples

Ongoing whole air sampling in flasks at several of the NEC sites by NOAA Earth System Research Laboratory’s Global Monitoring Division (NOAA/GMD) provides a check on the quality of the in situ measurements. The flasks are analyzed for CO_2_, CH_4_, and CO, among a suite of additional trace gases and isotopes that are not discussed here. The flask-sampling equipment draws air from one of the inlet lines at the top of the tower that is also shared by the continuous in situ measurement equipment (as indicated by the flask port in Fig. 4). The flask measurements are otherwise independent from the continuous in situ measurements. Flask samples at LEW and MSH are collected over a period of 10–30s (Sweeney et al., 2015; Andrews et al., 2014), while flask samples integrated over 1h are collected at TMD, NEB, NWB, and BWD (Turnbull et al., 2012) specifically as part of the Northeast Corridor project. All flask samples are taken in midafternoon local time (usually 19:00UTC). Comparisons at all the sites with available data indicate good agreement with little or no bias in the mean over the time period of the comparison, with the exception of CO at MSH, which shows a consistent bias with a median of 8 nmol mol^−1^, which is larger than the 1*σ* uncertainty assigned to either measurement (described in Sect. 4) and the standard deviation of the offsets themselves (Table 2). Target tank residuals for CO in this period range from 1 to 7 nmol mol^−1^, depending on the cylinders installed, indicating that at least some of this difference is caused by the calibration standard assigned value (possibly due to cylinder drift in time between the NOAA calibration and deployment to the site). Similar differences between NOAA flasks and in situ CO measurements were reported in Indianapolis (Richardson et al., 2017). This result requires further investigation, by sending the cylinders for recalibration and/or deploying different standards to the station. A significant bias in the CH_4_ offset at NWB is also apparent, at a mean of −5.5 nmol mol^−1^ but a median of −1.7 nmol mol^−1^, the result of a single outlier at −30 nmol mol^−1^ but with only 17 samples compared. BWD did not have any samples at the time of writing, thus we compare only LEW, MSH, TMD, NEB, and NWB.

Table 2 also reports the mean uncertainty, intended as a metric for comparison of the standard deviation of the offsets. For each flask sample, this uncertainty is the quadrature sum of the continuous data uncertainty (described in Sect. 4) at that hour, the standard deviation of the 1 min averages in the continuous data during that hour, and the uncertainty expected in the flask measurement, estimated here as 0.04 μmol mol^−1^ for CO_2_, 1.12 nmol mol^−1^ for CH_4_, and 0.59 nmol mol^−1^ for CO. The values for the flask uncertainty are from Table 1 in Sweeney et al. (2015), which reports the average offset between measurements of surface network and 12-pack flasks (such as those used for the NEC) filled with identical air after a short-term storage test. For CO_2_, flask offsets can be larger than indicated by those dry-air laboratory tests (Sweeney et al., 2015; Andrews et al., 2014; Karion et al., 2013), but we use 0.04 μmol mol^−1^ regardless because the average uncertainty in Table 2 is dominated by the atmospheric variability term and increasing the CO_2_ uncertainty in the flasks to 0.1 μmol mol^−1^ (for example) does not change the values significantly.

Standard deviations of the offsets (Table 2) show that there is quite a bit of scatter in the results, especially at the more urban sites that exhibit a lot of variability in the continuous data. For comparison, Turnbull et al. (2015) report agreement for CO_2_ between the same flask system and continuous in situ measurements in Indianapolis as 0.04 μmol mol^−1^ (mean) with a standard deviation of 0.38 μmol mol^−1^, somewhat smaller than what was observed at our sites. The standard deviation of offsets is usually lower than the average uncertainty, however, with the exception of CO_2_ at MSH and LEW, the two sites for which the flask samples are not integrated over an hour. It is likely that the large variability seen over an hour is the reason for the large scatter in the offsets. Because the continuous in situ measurements do not cover the entire hour of sampling (at the top level, the hourly average is typically the mean of only 40 min), the variability may not be captured in the mean uncertainty reported here and has a larger impact on the comparison than it would if the continuous hourly average was based on the full hour of observations. For example, a large plume or spike in concentration during a given hour might occur while the continuous system is sampling from the lower inlet and thus would not be included in the hourly average from the continuous system, while it would be included in the full 1h flask sample.

## 4 Uncertainty

The data set includes an uncertainty estimate on each hourly average data point, consistent with recommendations from the WMO (WMO, 2018). This uncertainty is our estimate of the uncertainty of the measurement itself and does not include atmospheric variability or assess the representativeness of the measurement of a true hourly mean.

### 4.1 Uncertainty of hourly mole fraction data

Verhulst et al. (2017) outlined a method for calculating an uncertainty in mole fraction measurements when using the single-tank calibration correction (drift correction). Here we present a brief overview but refer the reader to that paper for further details. All uncertainties are standard uncertainties, i.e., 1*σ* or *k* = 1. In the analysis below, we assume independent uncorrelated error components, given no evidence to the contrary and no physical reason to believe that they should be correlated; therefore, we sum the various components of the uncertainty in quadrature.

The uncertainty in the final mole fractions (*U*_air_) is expressed as the quadrature sum of several uncertainty components:

(7) $${\left(U_{\text{air}}\right)}^{2}={\left(U_{\text{extrap}}\right)}^{2}+{\left(U_{{\text{H}}_{2}\text{O}}\right)}^{2}+{\left(U_{\text{M}}\right)}^{2},$$

where $U_{{\text{H}}_{2}\text{O}}$ is the uncertainty due to the water vapor correction, *U*_M_ is a measurement uncertainty, and *U*_extrap_ is the uncertainty of the calibration fit when assigning values relative to a single standard tank (more detail on this can be found later in this section and in the following section). *U*_M_ encompasses errors due to drifting standard tank measurements (*U*_b_), short-term precision (*U*_p_), and error in the calibration standard’s mole fraction assignment by the calibration laboratory (*U*_scale_):

(8) $${\left(U_{\text{M}}\right)}^{2}={\left(U_{\text{p}}\right)}^{2}+{\left(U_{\text{b}}\right)}^{2}+{\left(U_{\text{scale}}\right)}^{2}.$$

Here we note that *U*_p_ for CO_2_ and CH_4_ is assigned as described by Verhulst et al. (2017), as the standard deviation of the individual measurements during each 1 min average during a calibration, but for CO it is assigned as the standard error (standard deviation divided by the square root of the number of samples in the mean), based on Allan variance tests (not shown) indicating that the precision of the CO measurement increases with the number of points used in the average. If no calibrations have been performed over an entire calendar year, *U*_p_ is set to the 10th percentile of the standard deviation of air measurements and *U*_b_ is set to a default value of 0.1 μmol mol^−1^, 0.5 nmol mol^−1^, and 4 nmol mol^−1^ for CO_2_, CH_4_, and CO, respectively. This default value for *U*_b_ is based on an upper limit of values that are observed in the network; typically, *U*_b_ is much smaller than these values (Verhulst et al., 2017). In the current data set, this has only occurred once: there were no calibrations run at MNC over the entire 2015 calendar year, but we have no knowledge of abnormal operations or changes during this period, with analyzer sensitivity being similar before and after this period.

Because these uncertainty components are also tested through the use of a target tank, or check standard, the uncertainty *U*_M_ is assigned as the root mean square of the target tank errors when those exceed the sum of the uncertainties above.

(9) $$U_{\text{M}}=U_{\text{TGT}}=\sqrt{\frac{\sum \left({\left(X_{\text{DC},\text{TGT}}-C_{\text{TGT}}\right)}^{2}\right)}{N}}$$

This residual is calculated by GCWerks, and the root-mean-square residual is interpolated in time as a moving 10d average. If a target tank has not been run through the system for 10d or longer, *U*_TGT_ is set to a default value that is currently set to 0.2 μmol mol^−1^, 1 nmol mol^−1^, and 6 nmol mol^−1^ for CO_2_, CH_4_, and CO, respectively, based on typical maximum values for this uncertainty calculated from many sites over several years. The target tank in the field generally has a concentration value very similar to the calibration tank, thus this residual is a good estimate of the uncertainty caused by the precision, baseline changes, and tank value assignment. However, it is not a good indicator of uncertainty at mole fractions different from that of the calibration tank. Therefore, we assign an added uncertainty component, *U*_extrap_, indicating the uncertainty that increases as the measurement value moves farther from the value of the calibration tank in the case of a single calibration standard. This was found to be a linear relationship for a series of similar model analyzers that were tested in a laboratory, and the uncertainty was described as follows:

(10) $$U_{\text{extrap}}=|\varepsilon |\left|X_{\text{DC,air}}-C_{\text{cal}}\right|.$$

See Verhulst et al. (2017) for details on determining the unitless slope of the uncertainty, epsilon (*ε*), which is currently assigned as 0.0025, 0.0031, and 0.0164 for CO_2_, CH_4_, and CO, respectively, for all data that are only drift corrected (i.e., not using a high standard).

### 4.2 Uncertainty for observations with additional standards available

**Figure 7.**
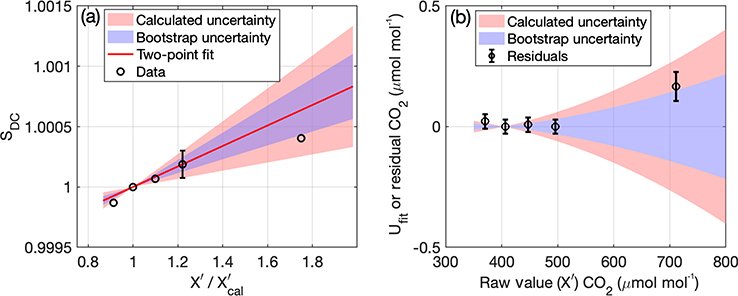
Uncertainty (1*σ*) in fit for two-point calibrations. (**a**) Two-point fit to drift-corrected sensitivity (*S*_DC_) (red line) with uncertainty (red shading) calculated using the uncertainty in the high standard (black circle with error bar). Blue shading shows uncertainty calculated using a bootstrap conducted by randomly selecting sets of two standards from the laboratory test (black circles) to calculate the slope. There is no uncertainty at 1 because the drift-corrected sensitivity is defined as equal to one at the value of the calibration standard. (**b**) Uncertainty in final CO_2_ as a function of raw CO_2_; red and blue shading have the same meaning as in (**a**).

When a high-standard tank is available and the secondary correction described in Sect. 3.4 is applied, the uncertainty analysis remains similar, but the uncertainty *U*_extrap_ from Eqs. (7) and (10) is replaced by an uncertainty in the two-point fit, *U*_fit_. To estimate this uncertainty for CO_2_ and CH_4_, we use the reported uncertainty of the assigned value of the high-standard and calibration-standard tanks, *U*_scale_, (typically 0.03 μmol mol^−1^ CO_2_ and 0.5 nmol mol^−1^ CH_4_ at 1*σ*) along with an estimate of the precision of the analyzer, *U*_p_, to estimate an uncertainty in the drift-corrected sensitivity of the high standard, *U*_SDC,hstd_, using standard propagation of errors (black error bar, Fig. 7a). We note that in the case where the value assigned to the high standard is through a propagation of the WMO scale at NIST, the assigned value has additional uncertainty; i.e., *U*_scale_ includes both the uncertainty that NOAA assigned to the cylinders used for the assignment and the uncertainty from the laboratory fit at NIST. This second uncertainty is equal to the standard deviation of the residuals of the fit and is added in quadrature to the NOAA uncertainty.

We note that the analysis described below assumes uncorrelated independent errors. We express the slope of drift-corrected sensitivity (*m*) and the overall drift-corrected sensitivity (*S*_DC_) as functions only of the drift-corrected sensitivity of the high standard, *S*_DC,hstd_:

(11) $$m=\frac{S_{\text{DC},\text{hstd}}-1}{X_{\text{hstd}}^{\prime }/X_{\text{cal}}^{\prime }-1}$$

(12) $$S_{\text{DC}}=m\left(\frac{X^{\prime }}{X_{\;\text{cal}}^{\prime }}-1\right)+1.$$

This second equation uses *b* = 1−*m*. Here we do not include uncertainty in the *x* coordinate, i.e., $X^{\prime }/X_{\text{cal}}^{\prime }$. Uncertainty in the slope is as follows:

(13) $$U_{m}=\left|\left(\frac{U_{\text{SDC},\text{hstd}}}{\left(X\prime_{\text{hstd}}/X\prime_{\text{cal}}\right)-1}\right)\right|.$$

We propagate the uncertainty in the drift-corrected sensitivity of the high standard, *U*_SDC,hstd_, to the overall drift-corrected sensitivity of all the air values using Eq. (14) and then to the two-point corrected air data by propagating through to obtain Eq. (15).

(14) $$U_{\text{SDC}}=U_{m}\left(X^{\prime }/X\prime_{\text{cal}}-1\right)\;\;=\left|\left(\frac{U_{\text{SDC},\text{hstd}}}{\left(X\prime_{\text{hstd}}/X\prime_{\text{cal}}\right)-1}\right)\left(X^{\prime }/X\prime_{\text{cal}}-1\right)\right|$$

(15) $$U_{\text{XSC},\text{air}}=U_{\text{fit}}=\left|\frac{U_{\text{SDC}}}{S_{\text{DC}}}\right|X_{\text{SC},\text{air}}$$

To evaluate the use of standard propagation of errors, we also use a bootstrap to estimate the uncertainty using the laboratory calibration shown in Fig. 6 by randomly selecting two tanks of the five tanks from the test to calculate 1000 versions of the correction (blue shading shows the standard deviation of the result, Fig. 7). For this test, the calculated 1*σ* uncertainty (red shading) was similar to the 1*σ* bootstrap uncertainty (slightly larger for CO_2_ and slightly smaller for CH_4_, not shown). This comparison indicates that the estimated uncertainty using the equations above compares reasonably well with the uncertainty we would derive from a bootstrap analysis, which gives us confidence in our methodology.

The uncertainty in *S*_DC_ leads to the estimate of the fit uncertainty, *U*_fit_, shown in Fig. 7b. To implement this uncertainty across all times and towers, we calculate it assuming a fixed nominal value of the high calibration standard of 490.50 μmol mol^−1^ CO_2_ and 2560.61 nmol mol^−1^ CH_4_. This is based on the value of the high standard that was in residence in the Earth Networks laboratory when several of the CRDS analyzers were tested and assigned two-point calibration corrections. We use the site-specific (instrument-specific and period-specific) slope and intercept that are applied to the data (which are static over the time period they are applied) and the value of the calibration tank to calculate the remainder of the values required for the uncertainty analysis.

Only one site so far, MSH, measures continuous CO, and the history of standard tanks there indicates significant uncertainty in tank value assignments with large target tank residuals and corresponding *U*_TGT_ relative to errors in slope. We have chosen not to implement the two-point calibration at this site for CO because the range of slopes of *S*_DC_ includes one, i.e., the correction is so small that the uncertainty dwarfs the correction.

Mean absolute residuals of the two-point fit for nine laboratory calibrations analyzed (seven tested at NOAA/ESRL and described by Verhulst et al., 2017, Table S2, and two additional units at NIST) average to 0.03 μmol mol^−1^ for CO_2_ between the calibration and high-standard data, and larger for the test that included an even higher-concentration tank, shown in Fig. 7 at ~ 711 μmol mol^−1^ for CO_2_. The fit uncertainty encompasses (at 1*σ*) this residual as well (Fig. 7b). The residuals at lower values can be explained by the uncertainty in the measurement (precision) and uncertainty in value assignment of the tanks. For CO, only eight tests were available, with a mean residual inside the range of the calibrations of 1.1 nmol mol^−1^, higher than the reported reproducibility from NOAA of 0.4 nmol mol^−1^ (all values are noted here at 1*σ* although they are given by NOAA at 2*σ*). This larger residual is likely caused by the lower precision of the analyzers for CO but also could be caused by larger uncertainty in the tank assignments, possibly due to drift in the mole fraction of the tanks themselves. We intend to conduct additional tests outside the two-point calibration range with additional analyzers and tanks to evaluate and possibly update this uncertainty component, *U*_fit_, as needed, and especially focus on CO if and when additional CO measurements are added to our network.

## 5 Network observations

Here we show some observations and time series of CO_2_ and CH_4_ from the NEC in situ tower network, focusing on data coverage, vertical gradients, and observed differences between urban and rural or outer suburban sites.

### 5.1 Data coverage and network expansion

**Figure 8.**
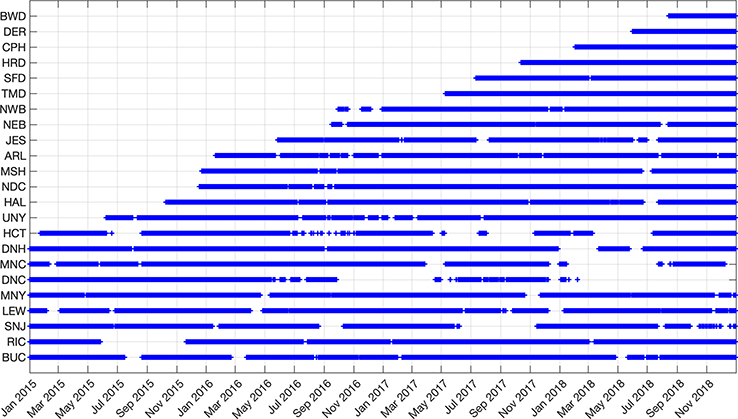
Data (CO_2_ and CH_4_, and CO for MSH only) availability from the various NIST-EN tower sites in the Northeast Corridor network included in this data release. Gaps represent data outages due to various failures (analyzer, communications, etc.).

The NEC network is continuously growing, with sites coming online at different times. Figure 8 shows the availability of hourly observations as the various sites have come online.

### 5.2 Vertical gradients

Observations in global trace gas measurement networks (e.g., AGAGE, GGRN) are specifically sited far from local sources or strong sinks to ensure that air reaching the site is representative of the large spatial scales of interest to a global study. This allows the observations to be more easily interpreted by a coarser global model (e.g., Peters et al., 2007). In urban networks, it is desirable to measure trace gas concentrations closer to sources so that finer spatial gradients can be used to inform emissions estimates at urban scales. However, a balance must be struck between the necessity to observe and distinguish sources that are in close proximity to each other and the ability of a transport and dispersion model to simulate the observations. In some instances, novel ways to simulate observations at low heights above ground level and in very dense networks have been used to resolve this problem (Berchet et al., 2017). In the NEC urban network in Washington, DC, and Baltimore, the tower sites were selected to be between 50 and 100m above the ground given the desire to place a tower in a specific location (as identified in an initial network design study by Lopez-Coto et al., 2017). Inlets at two (or three, at SFD) heights on the tower give some insight as to the proximity of each tower to sources whose emissions are not always vertically well mixed by the time they reach the inlets, depending on atmospheric stability conditions. Here we report average vertical gradients, determined using the observations at different levels, for the urban and background sites in our network. These gradients were calculated using hourly average data from each level, but because the instruments are only sampling from one level at any given time and cycling between them, there is an assumption of measurements averaged in a given hour being representative of the entire hour. Because different towers have different inlet heights and different vertical spacing between the lower and upper inlet, here we compare three urban sites (ARL, NDC, and JES) with inlets at similar heights, ~ 90m and ~ 50ma.g.l. We define the gradient as the mole fraction of CO_2_ or CH_4_ at the topmost inlet minus that of the lowermost inlet divided by the distance between them so that a negative gradient indicates a higher concentration at the lower inlet (the most common case).

**Figure 9.**
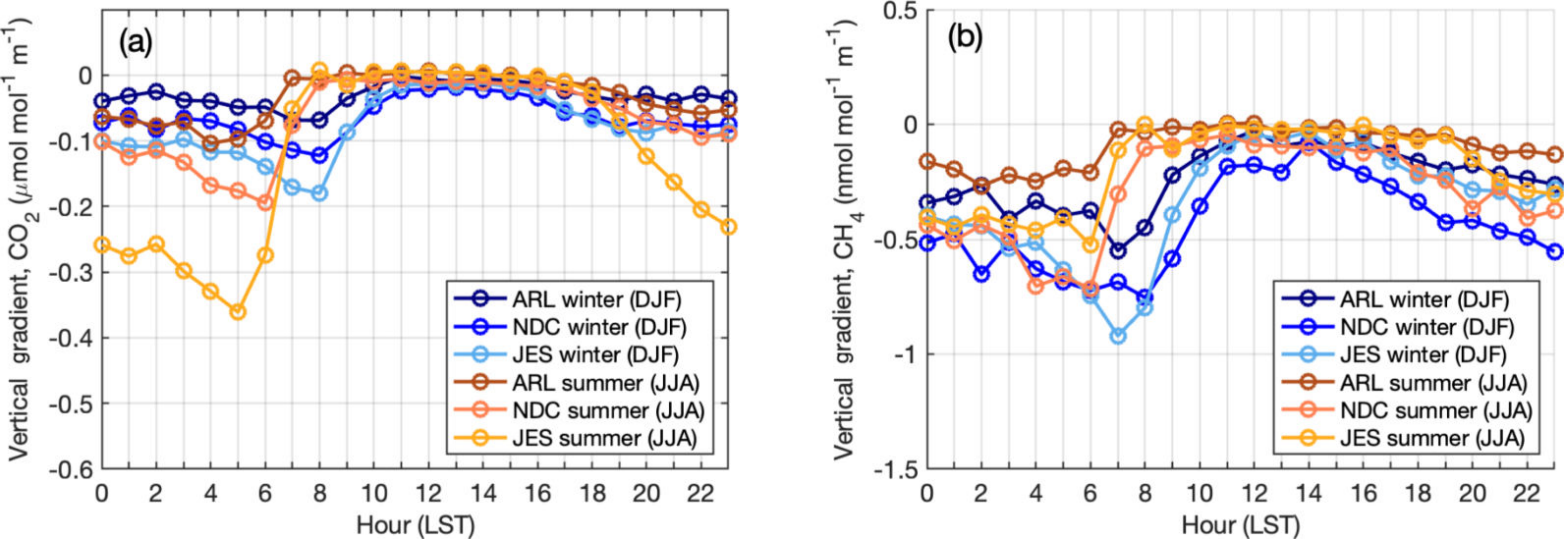
Diurnal cycle of vertical gradients in CO_2_ (**a**) and CH_4_ (**b**) for urban towers in the Washington–Baltimore area, averaged over 2015–2017 in winter (blue) and summer (orange), with shading indicating 1*σ* standard deviation among sites. Some of the spread can be caused by sampling in different years at the different sites. Sites included are HAL, ARL, NDC, NEB, NWB, and JES. HRD was excluded due to lack of data in this period.

Analysis of the diurnal cycle of the vertical gradient at urban sites in the Washington–Baltimore area (Fig. 9) indicates different characteristics in summer vs. winter. These differences are most likely caused by different meteorology and possible seasonal differences in timing of fluxes, especially for sites influenced by the urban biosphere. Greater turbulent mixing in summertime boundary layers and different timing in the boundary layer growth and collapse mostly dominate the seasonal differences. This analysis shows that at these three sites the wintertime average gradient in midafternoon hours (defined based on these figures as 11:00–16:00LST) is approximately −0.016 μmol mol^−1^ m^−1^ for CO_2_ (−0.105 nmol mol^−1^ m^−1^ for CH_4_), which translates to a −0.8 μmol mol^−1^ (−5.2nmol mol^−1^ for CH_4_) difference between levels spaced 50m apart; this is not an insignificant gradient. At other urban sites with shorter towers, they can be even larger. These observations can help evaluate vertical mixing in transport and dispersion models that might be used to estimate emissions, or to identify times when modeled and observed vertical gradients agree. Large vertical gradients overnight into the early morning at all sites and seasons are indicative of local sources (likely mostly anthropogenic but also including respiration from the biosphere) influencing the observations at these times when there is stable stratification in the boundary layer and concentrations are higher near the surface. The larger CO_2_ gradients overnight in summer compared to winter periods suggest a strong respiration signal at these urban sites, with a large degree of variability between sites indicated by large spread. Nighttime CH_4_ gradients are slightly larger in winter than summer, possibly reflecting greater wintertime anthropogenic CH_4_ emissions, or possibly due to seasonality in mixing layer heights.

**Figure 10.**
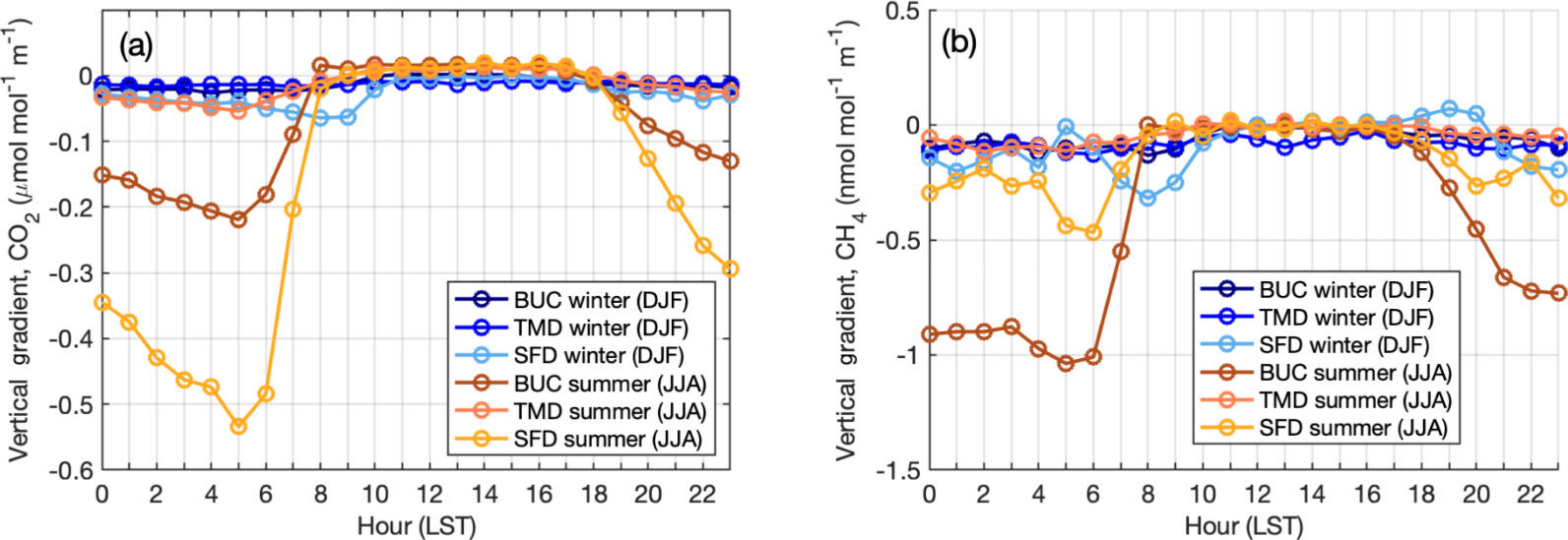
Diurnal cycle of vertical gradients in CO_2_ (**a**) and CH_4_ (b) at the three background towers for the Washington–Baltimore region in summer (orange shades) and winter (blue shades).

The diurnal cycle of the vertical gradients from the sites identified as background stations for the Washington–Baltimore urban network shows large variability in summertime gradients between the three stations (Fig. 10). Stafford, VA (SFD), shows that the surrounding biosphere causes relatively large gradients in nighttime and early morning hours at this low-density suburban site. These are apparent at Bucktown, MD (BUC), as well but less so at Thurmont, MD (TMD), a forested site in western Maryland. The large difference between summertime early morning vertical CO_2_ gradients at SFD and TMD, despite the similar surrounding land use (mostly deciduous forest, Fig. 3), might be caused by the elevation difference, as SFD is close to sea level while TMD is on a ridge at 561m elevation. BUC observations show larger CH_4_ gradients in summer, due to surrounding wetlands and agriculture (Fig. 3). Wintertime gradients are near zero at all hours at all three of these sites, indicating that they are far from local anthropogenic sources of either gas. We note that the top inlet height at BUC is lower, at 75m, than at SFD or TMD (100 and 111m), while the lower inlet is similar for all three (~ 50m). For SFD (inlets at 152, 100, and 50m), we use the 100 and 50m inlets to define the gradient to be more consistent with the inlet heights of the other towers (Table 1).

### 5.3 Urban and rural differences in seasonal cycles

**Figure 11.**
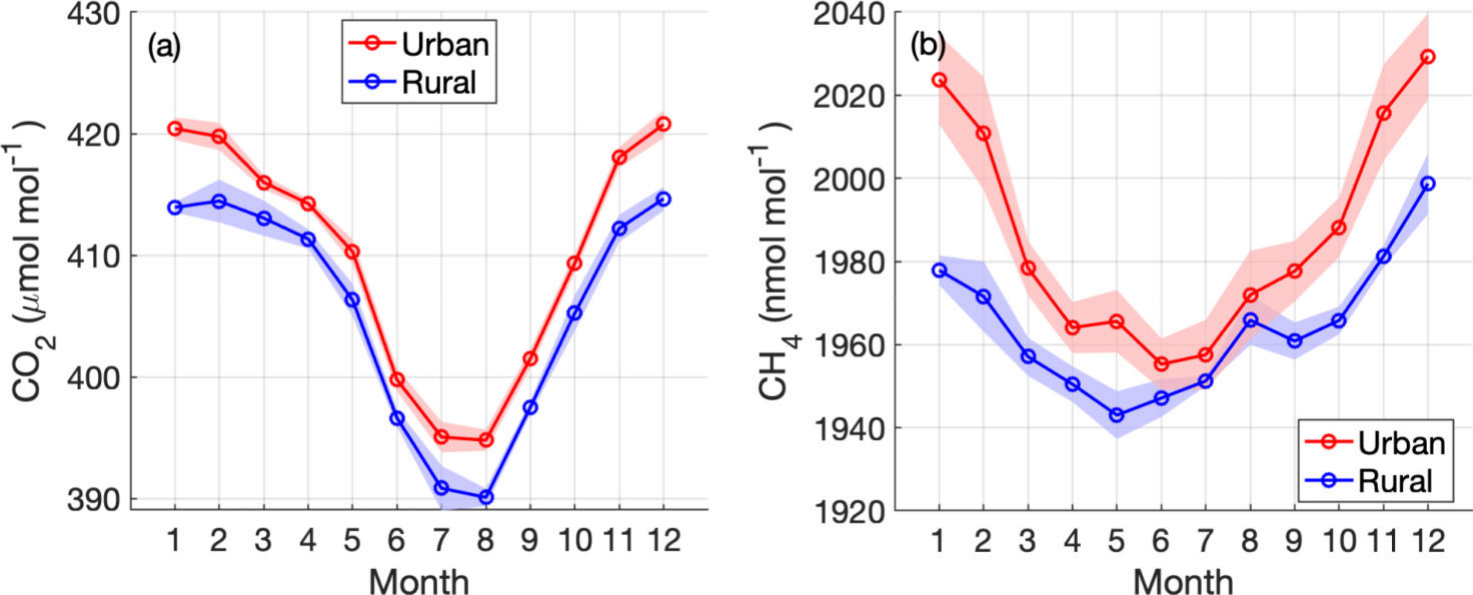
Seasonal cycles from urban and rural sites in the Washington, DC, and Baltimore region with at least 1 year of observations. Midafternoon (13:00–18:00 LST) daily averages are detrended using a linear fit to the annual trend at Mauna Loa (for CO_2_) and the global average (for CH_4_) (data from NOAA/ESRL) and then averaged monthly. Rural sites include TMD, SFD, and BUC; urban sites are ARL, NDC, JES, HAL, NEB, and NWB. Shading indicates 1 standard deviation of the averages from all the sites.

Here we continue to describe the network in terms of differences between rural (background) and urban stations, determining typical enhancements from urban influences. The seasonal cycles of CO_2_ and CH_4_ indicate enhancements in the urban sites in our network relative to the more rural stations throughout the year (Fig. 11). Summertime CH_4_ at urban sites is not as enhanced compared to the rural sites as it is in winter, possibly due to wetland sources influencing the background station at BUC or lower CH_4_ emissions from natural gas in urban areas. Similarly, for CO_2_, some of the rural stations surrounded by active vegetation (Fig. 3) are likely to show stronger influence from biospheric uptake than urban sites, especially in the summer months (Fig. 10). We specifically caution against using any of the in situ data from the NEC rural stations directly as a background for analysis of the urban enhancement without examining these issues. Sargent et al. (2018) indicate that for an analysis of CO_2_ enhancements in the Boston urban area, CO_2_ observations from upwind stations alone did not represent the correct background. Even when the air that reaches an urban tower originates near an upwind rural site, back trajectories (from a Lagrangian Particle Dispersion Model such as STILT, for example) indicate that much of the air may originate from a higher altitude than the upwind station. Thus, the measurement at an upwind station is not necessarily representative of the proper background or incoming concentration, given the large concentration gradients between measurements within the planetary boundary layer and in the free troposphere near background stations with local fluxes. Mueller et al. (2018) conducted an analysis of the issues concerning background determination for this urban network, mostly concerning the large emissions of both CO_2_ and CH_4_ upwind of the region that is difficult to capture at upwind stations. We will examine the proper background for investigating urban enhancements in the Washington, DC, and Baltimore, MD, area further in future work.

## 6 Data availability

This data set of hourly averaged observations from the Northeast Corridor tower-based network is available on the NIST data portal at https://data.nist.gov (last access: 23 March 2020) under the https://doi.org/10.18434/M32126 (Karion et al., 2019). Initially, the repository will contain data from 23 sites (Table 1) for years spanning 2015–2018; not all years are available for all sites. Files are version-dated, and the current plan is to provide annual updates for 2019 and beyond.

## 7 Conclusions

Here we present a data set of hourly average observations of CO_2_, CH_4_, and CO (where applicable) from a network of towers in the northeastern United States. Measurements are funded by NIST and conducted in a collaboration with Earth Networks, Inc., with quality control, assurance, and uncertainty determination conducted by a science team that includes NIST, Earth Networks, and collaborators from the Los Angeles Megacities Carbon Project from NASA/JPL and the Scripps Institution of Oceanography. We present 4 calendar years of data (2015 through 2018), with different stations coming online through the years, and most Washington, DC, and Baltimore, MD, urban stations becoming established after late 2015. We have also presented our methodology for calibrating the measurements to WMO scales for each gas and determining uncertainties for these measurements, as recommended by the WMO (WMO, 2018). We show that analysis of observations at two different inlet heights can be useful for determining the presence of emissions close to the towers, which may be necessary for evaluating the efficacy and choice of transport model used to analyze the data. We also note that the tower stations that were established to characterize incoming or background air are not necessarily appropriate for use directly as background for the urban stations, as they are often affected by local fluxes that do not influence the urban stations. A more careful treatment of incoming background air is necessary for any given analysis.

## Figures and Tables

**Table 1.** Northeast Corridor site list. Sites lacking location information are still in the planning phase, with no assigned site code or exact location.

Site Code EN site ID Location			Latitude	Longitude	Elev. (m)	Intake heights (m)	Measurements	Est. (mm/yyyy)
Northeast Corridor regional sites (12)								
DNC	GHG12	Danbury, NC	36.3769	−80.3689	703	100, 50	CO_2_, CH_4_	01/2015*
MNC	GHG15	Middlesex, NC	35.8313	−78.1453	74	213, 50	CO_2_, CH_4_	01/2015*
RIC	GHG18	Richmond, VA	37.5088	−77.5761	104	95, 50	CO_2_, CH_4_	01/2015*
SNJ	GHG19	Stockholm, NJ	41.1436	−74.5387	406	53, 42	CO_2_, CH_4_	01/2015*
HCT	GHG21	Hamden, CT	41.4337	−72.9452	204	100, 50	CO_2_, CH_4_	01/2015*
LEW	GHG25	Lewisburg, PA	40.9446	−76.8789	166	95, 50	CO_2_, CH_4_	01/2015*
DNH	GHG35	Durham, NH	43.7089	−72.1541	559	100, 50	CO_2_, CH_4_	01/2015*
UNY	GHG38	Utica, NY	42.8790	−74.7852	483	45, 35	CO_2_, CH_4_	01/2015*
MNY	GHG47	Mineola, NY	40.7495	−73.6384	34	90, 50	CO_2_, CH_4_	01/2015*
MSH	GHG54	Mashpee, MA	41.6567	−70.4975	32	46, 25	CO_2_, CH_4_, CO	12/2015
WNJ	GHG69	Waterford Works, NJ	39.7288	−74.8441	33	241, 201, 151, 98, 43	CO_2_, CH_4_	Planned 2020
		Philadelphia, PA					CO_2_, CH_4_	

Washington, DC, and Baltimore urban sites (12)								
HAL	GHG48	Halethorpe, MD	39.2552	−76.6753	70	58, 29	CO_2_, CH_4_	10/2015
ARL	GHG55	Arlington, VA	38.8917	−77.1317	111	92, 50	CO_2_, CH_4_	01/2016
NDC	GHG56	Northwestern DC	38.9499	−77.0796	128	91, 45	CO_2_, CH_4_	12/2015
NWB	GHG58	NW Baltimore, MD	39.3445	−76.6851	135	55, 27	CO_2_, CH_4_	09/2016
NEB	GHG59	NE Baltimore, MD	39.3154	−76.5830	44	67, 50	CO_2_, CH_4_	09/2016
JES	GHG60	Jessup, MD	39.1723	−76.7765	67	91, 49	CO_2_, CH_4_	05/2016
DER	GHG63	Derwood, MD	39.1347	−77.1419	125	54, 30	CO_2_, CH_4_	05/2018
CPH	GHG66	Capitol Heights, MD	38.8707	−76.8537	50	44, 25	CO_2_, CH_4_	02/2018
HRD	GHG67	Herndon, VA	38.9663	−77.3935	120	38, 27	CO_2_, CH_4_	10/2017
BWD	GHG64	Brentwood, MD	38.9343	−76.9556	17	51, 33	CO_2_, CH_4_	08/2018
BRK	GHG68	Burke, VA	38.7742	−77.2631	111	40, 24	CO_2_, CH_4_	Planned 2020
		Southeastern DC					CO_2_, CH_4_	

Washington, DC, and Baltimore background sites (5)								
BUC	GHG01	Bucktown, MD	38.4597	−76.0430	3	75,46	CO_2_, CH_4_	01/2015*
TMD	GHG61	Thurmont, MD	39.5768	−77.4881	561	113, 49	CO_2_, CH_4_	05/2017
SFD	GHG65	Stafford, VA	38.4459	−77.5300	77	152, 100, 50	CO_2_, CH_4_	07/2017
BVA	GHG62	Bluemont, VA	39.1379	−77.8326	486	111, 50	CO_2_, CH_4_	02/2020
		Delta, PA					CO_2_, CH_4_	

* If a station was established prior to the beginning of the project, its establishment date is listed as 01/2015, the start date of the project. Data prior to this date are not part of the NIST data release.

**Table 2.** Offsets (in situ – flask) between continuous in situ and NOAA/GMD flask measurements. CO_2_ offsets are reported in μmol mol^−1^, CH_4_ and CO are reported in nmol mol^−1^. Continuous in situ CO is only measured at MSH. The average uncertainty column for each gas indicates the 1*σ* uncertainty (summed in quadrature over flask uncertainty, in situ uncertainty, and atmospheric variability over the hour) averaged over the flask samples. See text for discussion.

Site	Number of flask samples	CO_2_ mean offset	CO_2_ median offset	CO_2_ standard deviation of offsets	CO_2_ mean unc.	CH_4_ mean offset	CH_4_ median offset	CH_4_ standard of offsets	CH_4_ mean unc.	CO mean offset	CO median offset	CO standard deviation of offsets	CO mean unc.
MSH	163	0.02	−0.02	0.65	0.43	−0.2	−0.2	2.3	2.1	−9.0	−8.2	5.9	6.0
LEW	315	0.01	−0.07	0.88	0.68	1.1	0.4	8.4	7.8	-	-	-	-
TMD	80	0.17	0.15	0.51	0.69	0.0	0.5	5.5	5.2	-	-	-	-
NEB	32	−0.09	0.08	0.75	1.02	−0.5	0.6	6.8	13.4	-	-	-	-
NWB	17	−0.014	0.02	0.73	0.99	−5.5	−1.7	9.8	8.3	-	-	-	-

## Footnotes

1 Certain commercial equipment, instruments, or materials are identified in this paper in order to specify the experimental procedure adequately. Such identification is not intended to imply recommendation or endorsement by the National Institute of Standards and Technology nor is it intended to imply that the materials or equipment identified are necessarily the best available for the purpose.
